# Comparison of Intraocular Pressure before and after Laser In Situ Keratomileusis Refractive Surgery Measured with Perkins Tonometry, Noncontact Tonometry, and Transpalpebral Tonometry

**DOI:** 10.1155/2015/683895

**Published:** 2015-06-08

**Authors:** Isabel Cacho, Juan Sanchez-Naves, Laura Batres, Jesús Pintor, Gonzalo Carracedo

**Affiliations:** ^1^Instituto Balear de Oftalmología, 07011 Palma de Mallorca, Spain; ^2^Departamento de Óptica II (Optometría y Visión), Facultad de Óptica y Optometría, Universidad Complutense de Madrid, 28037 Madrid, Spain; ^3^Departamento de Bioquímica y Biología Molecular IV, Facultad de Óptica y Optometría, Universidad Complutense de Madrid, 28037 Madrid, Spain

## Abstract

*Purpose*. To compare the intraocular pressure (IOP) before and after Laser In Situ Keratomileusis (LASIK), measured by Diaton, Perkins, and noncontact air pulse tonometers.* Methods*. Fifty-seven patients with a mean age of 34.88 were scheduled for myopia LASIK treatment. Spherical equivalent refraction (SER), corneal curvature (K), and central corneal thickness (CCT) and superior corneal thickness (SCT) were obtained before and after LASIK surgery. IOP values before and after surgery were measured using Diaton, Perkins, and noncontact air pulse tonometers.* Results*. The IOP values before and after LASIK surgery using Perkins tonometer and air tonometers were statistically significant (*p* < 0.05). However, no significant differences were found (*p* > 0.05) for IOP values measured with Diaton tonometer. CCT decreases significantly after surgery (*p* < 0.05) but no statistical differences were found in SCT (*p* = 0.08). Correlations between pre- and postsurgery were found for all tonometers used, with *p* = 0.001 and *r* = 0.434 for the air pulse tonometer, *p* = 0.008 and *r* = 0.355 for Perkins, and *p* < 0.001 and *r* = 0.637 for Diaton.* Conclusion*. Transpalpebral tonometry may be useful for measuring postsurgery IOP after myopic LASIK ablation because this technique is not influenced by the treatment.

## 1. Introduction

Intraocular pressure (IOP) measurement is necessary for the screening and diagnosis of glaucoma as well as being an inclusion/exclusion criterion for all types of ocular surgical procedures. LASIK (Laser In Situ Keratomileusis) is the most common surgical technique used to correct low and moderate refractive errors. In the case of myopia, this technique causes an ablation of the corneal tissue that induces changes in the corneal curvature, central corneal thickness (CCT), and corneal rigidity. These changes alter the postsurgical measurement of the IOP (intraocular pressure) measured with Goldmann applanation (GAT) or noncontact air tonometry [1]. There is evidence that the central corneal ablation causes a constant decrease in the tonometry values of around 1.6 mmHg (in myopes and hyperopes as well), and myopic patients exhibit an additional reduction of IOP readings due to the fact that the maximum tissue ablation is in the very center of the cornea, which is 0.029 ± 0.003 mmHg per micrometer of ablated tissue, that is, if we consider a mean corneal tissue remove of 15 microns per diopter, for every diopter we see, and underestimate readings of 0.5 mmHg [2].

Intraocular pressure is a significant risk factor in the diagnosis of glaucoma. Therefore, in patients treated with LASIK, the IOP measurement may be lower and this would lead to later detection of glaucoma [3].

Glaucoma is distinguished from other optic neuropathies by slow progression over months to years. The prevalence is low before 40 years of age and increases exponentially with age [4]. The association between myopia and glaucoma has been reported by authors from different countries [5–7], who claim that high myopia is a predisposing factor for glaucoma [8].

Some recent studies on LASIK surgery for myopia correction reported that patients experienced an increase in the intraocular pressure during flap creation that decreased to normal when the suction ended [9]. Furthermore, it has been reported that postsurgical variations in corneal biomechanics lead to complications such as myopia regression. Understanding the biomechanical properties to prevent the onset of these complications has led to a lot of research [10, 11].

The Perkins applanation tonometer is a portable version of the Goldmann Applanation Tonometer (GAT). GAT is a gold standard method for the measure of IOP; however, the clinician should be careful when interpreting the measurements in 2002; Bhan et al. [12] showed a limitation: GAT underestimates IOP in eyes with significantly thinner corneas and overestimates it with thicker corneas. Its accuracy is influenced by corneal thickness, curvature, rigidity, and corneal hydration [13, 14]. Although it is true that in eyes with increased CCT this measuring technique tends to overestimate IOP, the same seems to be valid for corneas that are flatter or steeper than usual [15]. Several studies have shown that the biomechanical corneal disorder produced by the flap creation for LASIK treatment affects the postsurgical IOP measurement by Goldmann applanation and air tonometry [16, 17].

The Diaton tonometer measures the intrapalpebral IOP by exerting pressure on the peripheral cornea, outside the ablation, and on the sclerocorneal limbus. The thickness of this peripheral area is not affected after myopia LASIK surgery [18]. The noncontact air tonometry is an applanation method not requiring anesthesia because it uses a standardized blow of air to flatten the cornea. The flattening is applied to the centre of the cornea and the blow increases its intensity until the flattening of the cornea is obtained; therefore, the higher the intensity, the higher the IOP reading [19].

The aim of this study was to compare the intraocular pressure measured by three different tonometers before and after LASIK surgery. Two of them perform the measurement in the centre of the cornea and the other one in the corneal periphery.

## 2. Methods

### 2.1. Patients

Fifty-seven patients with ages ranging from 22 to 53 (average 34.88 ± 8.86) were scheduled for LASIK to treat myopia. One eye per patient was selected at random. More detailed demographic characteristics of the population are shown in Table 1.

Patients were subjected to a complete presurgical ophthalmic examination that includes the IOP tests for this study. These IOP tests were repeated one month after surgery.

The study was conducted in compliance with good clinical practice guidelines, informed consent regulations, and the tenets of the Declaration of Helsinki (WMA, 2013) [20]. The study was approved by the Balearic Institute of Ophthalmology IRB. All the subjects enrolled in the study were adults older than 18 years who were able to give informed consent and they could leave the study at any time.

### 2.2. Clinical Measures

Before and after LASIK surgery, spherical equivalent refraction (SER), corneal curvature (K), and central corneal thickness (CCT) and superior corneal thickness (SCT) were obtained. IOP values pre- and postsurgery were measured using three different techniques: Diaton tonometer, Perkins tonometer, and air tonometer.

### 2.3. Surgery Procedures

Surgery was performed by the same surgeon (Juan Sanchez-Naves) using the Technolas 217 flying spot excimer laser system, version V 312.383 (Bausch & Lomb, Irvine, CA, USA). Laser parameters included the following: wavelength of 193 nm, radiant exposure (Fluence) of 160 mJ/cm^2^, pulse repetition rate of 50 Hz, average ablation depth/pulse of 0.25 *μ*m on the cornea, and an ablation zone diameter from 6.5 to 7 mm with a transition zone of 0.5 mm. The XP automated microkeratome (Bausch & Lomb, Irvine, CA, USA), a superior-hinged corneal flap (120 or 140 lm head plates), was created. Patients were prescribed topical antibiotic and steroid drops (Tobradex, Alcon, TX, USA) every 6 hr for 5 days. For all eyes, presurgical manifest refraction was selected as the target correction.

### 2.4. IOP Measurement

Three measurements were taken on each patient and the average of the readings was recorded as the final IOP. During the measurement, subjects were asked to keep the eye open and fixate into the distance behind the examiner. First, three consecutive measurements of IOP with noncontact tonometer, based on air puff (Topcon CT60, Topcon Corporation, Tokyo, Japan), were performed with an approximate time interval of 3 seconds. The IOP measurements were taken by means of a Perkins tonometer after instillation of 1 drop of double anaesthetic Colircusí which contains tetracaine 0.1% and oxybuprocaine 0.4% (Colircusí, Alcon Cusí SA, Barcelona). Finally, ten minutes later, Diaton tonometer (Ryazan State Instrument Making Enterprise, Ryazan, Russia) measurements were performed in the sitting position with the patient gazing at a 45° angle, placing the tonometer in contact with the eyelid margin at the superior limbus. The device was activated when the signalling mechanism indicated the correct vertical position. There was a 5-minute interval between the Perkins and Diaton measurements. Central and peripheral corneal thicknesses at 4.5 mm superior location from the centre of the cornea (CCT and SCT) were measured, calculated, and provided by videokeratography (Orbscan II, Bausch & Lomb, Rochester, New York, USA).

### 2.5. Statistical Analysis

Data were analyzed by statistical package SPSS version 17.0 for Windows (SPSS, Inc., Chicago, IL). The values presented are the means ± SD of the values obtained. Normality of distribution was assessed using the Shapiro-Wilks test. The differences between pre- and postsurgery IOPs for each tonometer measurement and the differences between CCT and peripheral corneal thicknesses at superior location were tested for statistical significance using the Student paired* t*-test. The IOPs between different tonometry devices were compared with Student's* t*-test for independent samples. Correlations between measurement before and after surgery were evaluated using a Pearson correlation test.

The spherical equivalent refraction (SER) was calculated as the sum of the sphere and half the refractive astigmatism in dioptres (D) obtained after standard subjective refraction. Linear regression analysis was used to quantify the correlation in IOP measurements and various parameters: change in keratometry, change in dioptres of SER, and changes with the age. *p* < 0.05 was considered statistically significant.

## 3. Results

**Figure 1:**
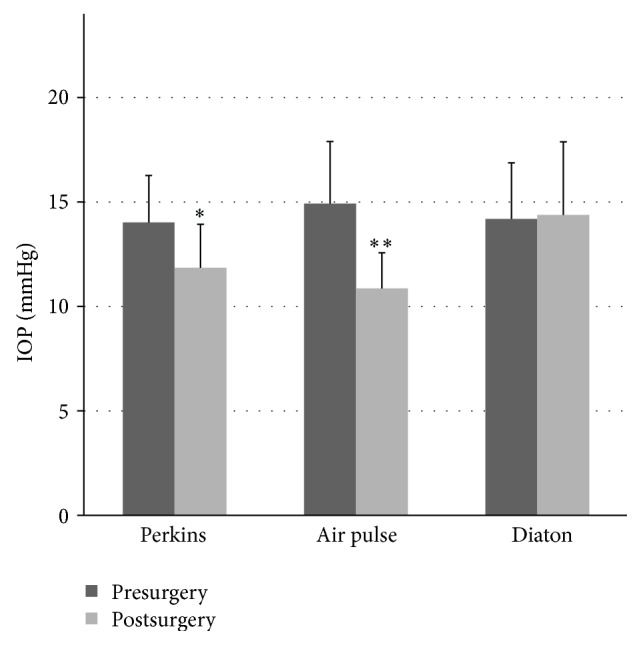
Comparison of the pre- and postsurgery IOP for the Perkins, noncontact, and transpalpebral tonometries (^*∗*^ *p* < 0.05 Diaton versus Perkins; ^*∗∗*^ *p* < 0.05 Diaton versus air pulse tonometry; Student's* t-*test for independent samples).

The differences of the IOP values between pre- and post-LASIK surgery measured with the Perkins and air tonometers were statistically significant (*p* < 0.05). However, no significant differences were found (*p* > 0.05) in IOP values pre- and post-LASIK surgery measured with Diaton tonometer (Figure 1). Regarding corneal thickness, CCT decreases significantly after surgery (*p* < 0.05) but no statistical differences were found in SCT (*p* = 0.08).  Table 2 shows the mean of IOP with each tonometer and the pre- and postsurgery corneal thicknesses.

Correlations between pre- and postsurgery were found for all tonometers, with *p* = 0.001 and *r* = 0.434 for air pulse tonometer, *p* = 0.008 and *r* = 0.355 for Perkins tonometer, and *p* < 0.001 and *r* = 0.637 for Diaton tonometer. The CCT and SCT values taken pre-and post-LASIK surgery showed a positive correlation of *p* < 0.001 and *r* = 0.626 and *p* = 0.001 and *r* = 0.542, respectively.

The IOP values using the air tonometer and the Perkins tonometer were correlated both before surgery with *p* = 0.002 and *r* = 0.407 and after surgery with *p* = 0.002 and *r* = 0.408, although no correlation was found between the IOP values measured with Diaton tonometer and Perkins tonometer before with *p* = 0.338 and *r* = 0.132 and after surgery with *p* = 0.358 and *r* = 0.124.

Regarding corneal thickness, CCT values were found to correlate with the IOP values measured using Perkins and air tonometers, both before surgery with *p* = 0.035 and *r* = 0.286, and *p* = 0.004 and *r* = 0.373 and after surgery with *p* = 0.017 and *r* = 0.312, respectively. However, the SCT values for both the pre- and the postsurgery measurements did not correlate with the IOP values from the Diaton tonometer with *p* = 0.369 and *r* = 0.124, and with *p* = 0.453 and *r* = 0.167, respectively.

Finally, the change in diopters for the SER before surgery was correlated with the difference between the IOP values measured before and after surgery using air tonometer with *p* = 0.009 and *r* = −0.343 and also with the CCT with *p* < 0.001 and *r* = −0.660. However, no correlation was found with Perkins and Diaton tonometer with *p* = 0.256 and *r* = −0.156, and with *p* = 0.466 and *r* = −0.102, respectively.

## 4. Discussion

The aim of this study was to evaluate the IOP before and after myopic LASIK surgery taking into account the implication that corneal thickness has on the measurement. The accuracy of intraocular pressure measurement is critical for the glaucoma diagnosis and its follow-up. Low IOP readings after LASIK would result in a delayed diagnosis of glaucoma or recognition of ocular hypertensive patients [21]. Various measurement methods have been used previously for the quantification of IOP [22]. The Goldmann applanation tonometry is accepted as the gold standard in IOP measurement but it seems that central corneal thickness is an important factor in this measurement overestimating the IOP on thick corneas and underestimating it on thin corneas [15]. On the other hand, there is evidence that noncontact tonometry gives higher readings than Goldmann's, particularly in adult patients. This tonometry is also dependent on central corneal thickness [23]. Regarding transpalpebral tonometry, there is discrepancy between authors on the accuracy of the instrument [24, 25]. Sandner et al. [26] found a sufficient correlation between Goldmann and transpalpebral tonometry, concluding that Diaton may be a good tool for screening. However, other authors, did not find this correlation, probably for a substantial interexaminers variation [27]. In our study, no correlation was found between Perkins tonometry and transpalpebral tonometry, probably due to a lack of reliability, described by others authors [25, 28].

Our results showed that readings obtained with the Perkins and air tonometers, measuring the IOP in the center of the cornea and therefore in the ablation zone, were significantly lower after the refractive surgery when compared to the presurgery values. However, the transpalpebral tonometer, which takes the IOP in the superior zone of the cornea, showed the same values before and after surgery. It seems that the cause for this IOP decrease may be the central corneal thinning resulting from the surgery together with the biomechanical change of the cornea after the flap creation. Similarly, Shemesh et al. [29] found that patients undergoing LASIK and LASEK treatments showed lower IOP after refractive surgery when measured with Goldmann applanation tonometry but not when measured with dynamic contour tonometry, which is apparently independent of central corneal thickness. Also, Shousha et al. [30] concluded that IOP lowered after LASIK and epiLASIK treatments when measured with both Goldmann and noncontact tonometry.

It could seem surprising the no correlation between SER before surgery and Perkins tonometry. This correlation is dependent on corneal thickness,* K* values, biomechanical characteristics of the cornea, and ablation diameter but it has been described that IOP after surgery is only dependent on corneal thickness and* K* values, obtaining inaccurate IOP measures [1, 31]. This fact could be explaining the lack of correlation between Perkins tonometry and SER before surgery.

The main limitation of transpalpebral tonometer is inaccuracy in elevated intraocular pressure eyes, as there is evidence that Diaton underestimates the intraocular pressure measurement when compared to the gold standard Goldmann tonometry [32]. More research is needed to validate the new methods to obtain an IOP reading nondependent on central corneal thickness, as the Diaton tonometer for glaucoma patients. Another limitation of the study is the difficulty to calculate the thickness on the limbus, exactly in the Diaton point of measure. In our study, SCT was measured at 4.5 mm of the central cornea to justify the no change in the peripheral thickness, but this is not exactly the point that the transpalpebral tonometer does the measurement. The no correlation between SCT and Diaton tonometry indicates that lid biomechanics and thickness have an important role and therefore more studies about this topic would be necessary.

In conclusion, the transpalpebral tonometer may be useful to control the IOP after LASIK surgery as it does not depend on the ablation and thinning of the central cornea after myopic refractive surgery.

## Figures and Tables

**Table 1:** Demographic characteristics of the participants in the study.

Parameter	LASIK	
Number of eyes (patients)	57 (57)	
Age (years) (mean (SD))	34.88 (8.86)	
Age range (years)	[22, 53]	
Gender (male/female)	[27, 30]	
Axial length (mm) (mean (SD))	24.76 (0.88)	
Sphere presurgery (D) (mean (SD))	−2.99 (1.24)	
Cylinder presurgery (D) (mean (SD))	−0.56 (0.40)	
	91.98 (59.58)	

	Presurgery	Postsurgery

Flat (D) (mean (SD))	43.44 (1.47)	40.92 (1.32)^*∗*^
Steep (D) (mean (SD))	43.62 (1.42)	41.12 (1.54)^*∗*^

^*∗*^Presurgery versus postsurgery. *p* < 0.05. Student's paired *t*-test. For details see Section 2.

**Table 2:** Intraocular pressure readings with Perkins, air and Diaton tonometers, and central and superior pachymetries before and after LASIK.

Parameter	Presurgery	Postsurgery	Presurgery − postsurgery	*p* value
IOP Perkins (mmHg) (mean (SD))	14.02 ± 2.25	11.85 ± 2.08	2.16 ± 2.47	*p* < 0.001^*∗*^
IOP air tonometry (mmHg) (mean (SD))	14.92 ± 2.98	10.86 ± 1.71	4.05 ± 2.72	*p* < 0.001^*∗*^
IOP Diaton (mmHg) (mean (SD))	14.18 ± 2.70	14.38 ± 3.50	−0.20 ± 2.73	0.590
Central pachymetry (*µ*m) (mean (SD))	572.59 ± 45.41	475.91 ± 55.95	96.67 ± 44.83	*p* < 0.001^*∗*^
Superior pachymetry (*µ*m) (mean (SD))	658.62 ± 31.69	645.18 ± 29.41	13.44 ± 29.30	0.080

^*∗*^Presurgery versus postsurgery. *p* < 0.05. Student's paired *t*-test.
